# Facial Pyoderma Gangrenosum Revealing Inflammatory Bowel Disease After Prior Total Colectomy

**DOI:** 10.7759/cureus.95332

**Published:** 2025-10-24

**Authors:** Maggie S Sanders, Andrew T Kosa, David G Cotter

**Affiliations:** 1 Dermatology, Kirk Kerkorian School of Medicine at University of Nevada, Las Vegas, USA; 2 Dermatology, Las Vegas Dermatology, Las Vegas, USA

**Keywords:** colectomy, extraintestinal manifestations in inflammatory bowel disease, inflammatory bowel disease, neutrophilic dermatoses, pathergy, pyoderma gangrenosum, thread lift, ulcer

## Abstract

Pyoderma gangrenosum (PG) is a rare neutrophilic dermatosis associated with systemic conditions such as inflammatory bowel disease (IBD) and hematologic malignancy. Lesions classically present on the lower extremities and rarely arise on the face. We report an unusual case of facial PG that developed at the site of a prior cosmetic thread lift. The lesions ultimately led to a diagnosis of underlying IBD despite the patient’s history of a total colectomy following complications from a non-IBD related procedure. This case highlights that clinicians must always evaluate for underlying conditions in patients with PG, even when symptoms are absent or surgical history is misleading.

## Introduction

Pyoderma gangrenosum (PG) is a rare inflammatory dermatologic condition that presents with rapidly progressing cutaneous ulcers. PG is classified as a neutrophilic dermatosis (ND), a group of cutaneous diseases characterized by lesions with neutrophilic infiltrates on histology in the absence of infection [1]. While the exact underlying cause is unknown, PG is hypothesized to be an autoinflammatory disease [1,2]. While some cases of PG are idiopathic, a strong association has been established between PG and various disorders, including inflammatory bowel disease (IBD), inflammatory arthritis, hematologic malignancy, and rheumatologic conditions [2,3]. Lesions typically affect the lower limbs and are rarely seen on the face [2]. Some reports have documented PG developing at sites of prior surgical trauma [4,5].

We present a novel case of facial PG in a patient with a remote history of a permanent thread lift, which ultimately revealed a new diagnosis of IBD years after undergoing a total colectomy due to a complication of spinal surgery.

## Case presentation

A 75-year-old woman with a history of chronic non-healing lower leg wounds presented to our clinic for evaluation of ulcers on the left cheek and left leg. She reported having undergone a thread facelift 30 years prior and stated that a suture had recently begun protruding through the skin, leading to ulceration. The patient had been self-treating the wound with clobetasol and mupirocin ointments without improvement.

**Figure 1:**
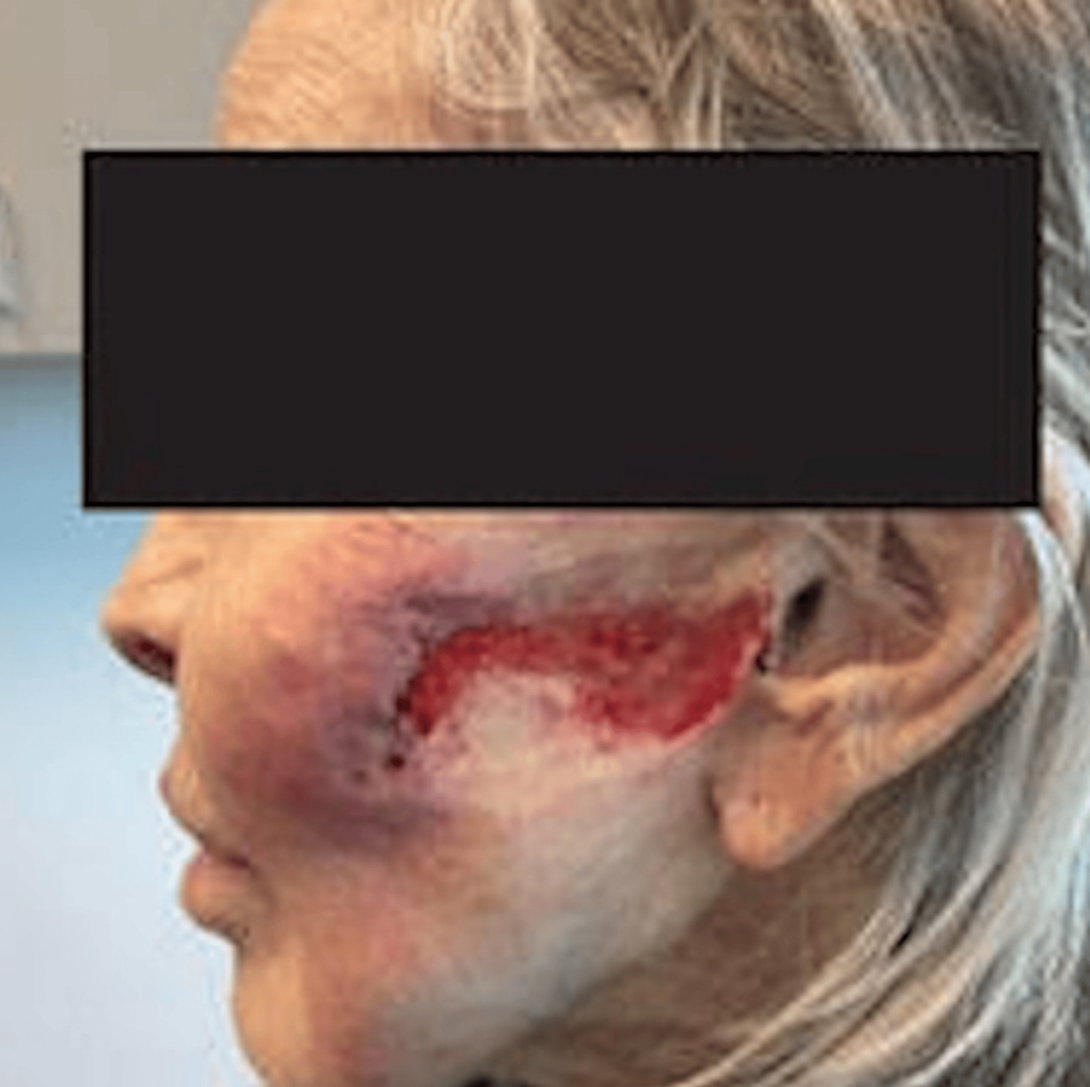
Superficial, irregularly shaped wound of the left cheek with rolled borders at the site of a previous thread facelift.

Examination revealed an irregularly shaped, superficial ulcer with areas of rolled, violaceous borders on the left cheek (Figure 1), as well as a similar lesion on the left leg. The patient's previous history of chronic, non-healing ulcers raised suspicion for pyoderma gangrenosum (PG). Biopsies of prior lower extremity wounds showed non-specific neutrophilic inflammation, further supporting a diagnosis of PG. Her complete blood count, complete metabolic panel, and complete review of systems, including any current or past gastrointestinal (GI) symptoms suggestive of IBD, were negative. However, the patient underwent a total colectomy years prior due to complications during surgical removal of a spinal cord meningioma. Since her total colectomy may have mitigated any GI symptoms before they could arise, serologic assessment for markers of IBD were ordered. Laboratory workup returned positive for perinuclear antineutrophil cytoplasmic antibodies (p-ANCA), myeloperoxidase antibody, and anti-saccharomyces cerevisiae antibody (ASCA). While awaiting wound culture results, she was prescribed azithromycin due to an inability to tolerate gastrointestinal side effects of other antibiotics.

**Figure 2:**
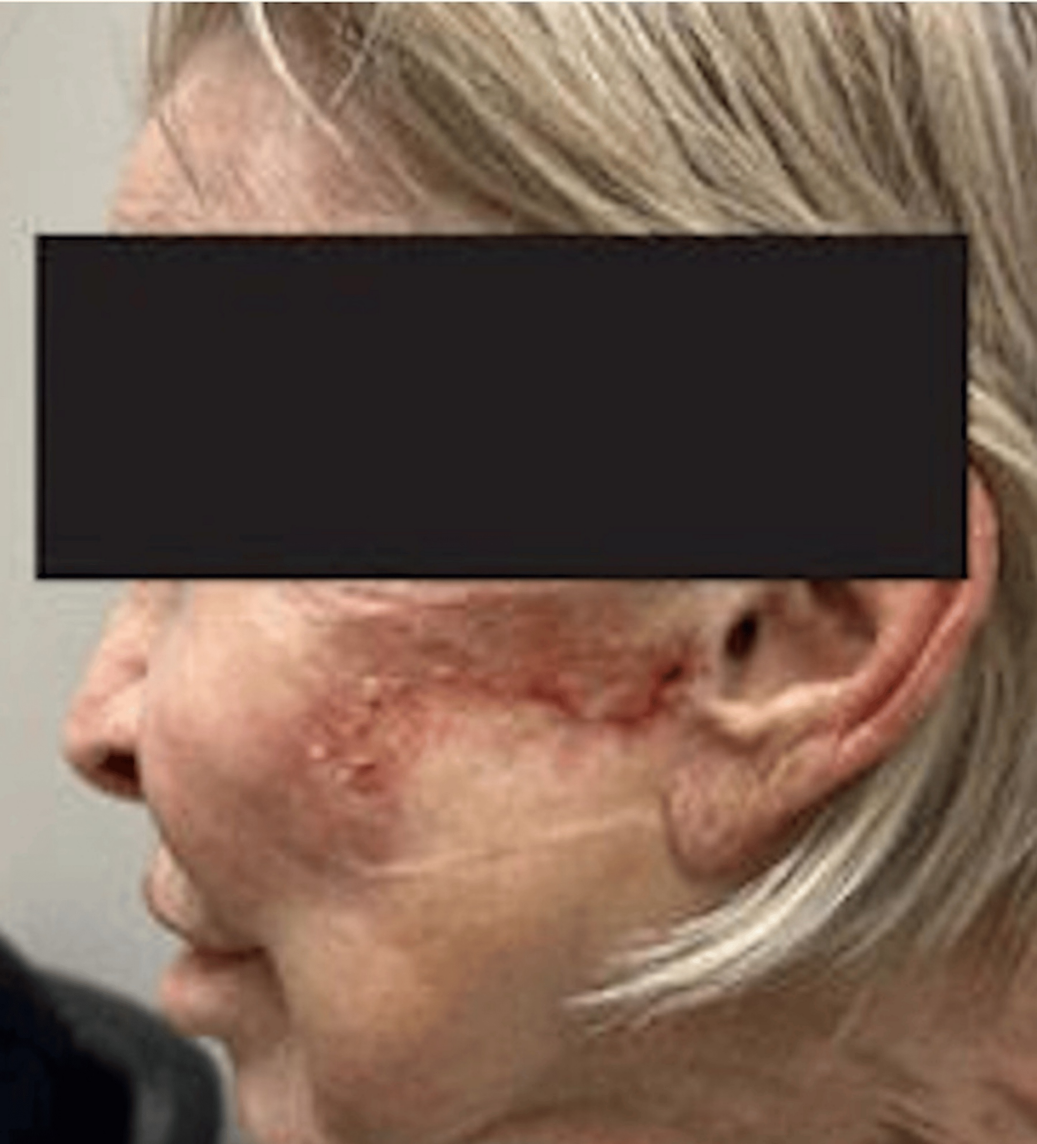
Left cheek wound after a two-month prednisone taper and topical clobetasol 0.05% ointment.

PG was the favored diagnosis in the setting of this patient's history of recurrent non-healing ulcers, the clinical appearance of the facial lesion, positive IBD markers, and negative wound cultures. The patient was started on prednisone 40 mg daily, topical clobetasol 0.05% ointment, and mupirocin. After one month of treatment, the facial lesion showed significant improvement and she was started on a prednisone taper. After two months of treatment, the facial ulcer fully healed (Figure 2).

## Discussion

Facial PG is rare, with only a limited number of cases reported in the literature. In a small study of 170 patients with PG, only 5 (2.9%) had lesions involving the head [6]. Of these, fewer than half had associated systemic conditions such as IBD and monoclonal gammopathy of undetermined significance (MGUS) [6].

In this case, we suspect that pathergy from the migration of the patient's past thread facelift triggered PG in the setting of an underlying and undiagnosed IBD-related inflammatory milieu. Although she lacked gastrointestinal symptoms, positive serologies ultimately revealed a likely underlying diagnosis of IBD. Her prior total colectomy likely prevented typical IBD symptoms, leaving her probable PG as the sole clinical clue to her underlying condition. Notably, given a lack of surgical pathology from the patient's remote colectomy, IBD cannot be definitively diagnosed; however, suspicion is high given positive serologies. Therefore, idiopathic PG cannot be entirely ruled out in this case. The patient has since been asymptomatic without any recurrence; thus, maintenance therapy with off-label targeted agents such as adalimumab was not deemed necessary. The patient will continue to be followed, and steroid-sparing agents may be considered in the event of recurrence. 

It has been hypothesized that surgical treatment of IBD, such as colectomy, may alleviate extraintestinal manifestations (EIM). One study of 121 patients found that colectomy successfully treated EIM in 45% of cases [7]. However, our case supports the idea that colectomy alone does not preclude the future development of EIMs such as PG.

Emerging evidence underscores the utility of fecal calprotectin as a marker for subclinical inflammation in IBD-associated PG. One recent retrospective cohort study found that higher levels of fecal calprotectin correlated with PG severity and increased disease activity [8]. Additionally, it has been shown that positive fecal calprotectin (>150 micrograms/gram) in cases of idiopathic PG may be a predictor of eventual IBD diagnosis [8,9].

## Conclusions

This case is particularly unusual due to facial involvement, as PG most often affects the lower extremities and rarely occurs on the head or neck. The delayed reemergence of an old thread lift suture suggests that pathergy likely played a central role in triggering the lesion at this site. These factors, combined with the absence of gastrointestinal symptoms, made the diagnosis of PG especially challenging. This case also reinforces that PG can be the first clinical clue to an underlying systemic disease such as IBD, even in patients who are asymptomatic or have undergone a colectomy. Documenting presentations such as this case broadens awareness of different presentations of PG, underscores the role of pathergy, and highlights the need to consider systemic disease even in atypical scenarios.
